# Enhanced Anticancer Activity of 5’-DFUR-PCL-MPEG Polymeric Prodrug Micelles Encapsulating Chemotherapeutic Drugs

**DOI:** 10.3390/nano8121041

**Published:** 2018-12-13

**Authors:** Alicia J. Sawdon, Jun Zhang, Xutu Wang, Ching-An Peng

**Affiliations:** 1 Department of Chemical Engineering, Michigan Technological University, Houghton, MI, 49931, USA; ajsawdon@gmail.com; 2 Department of Biological Engineering, University of Idaho, Moscow, ID 83844, USA; zhan8177@vandals.uidaho.edu (J.Z.); wang3213@vandals.uidaho.edu (X.W.)

**Keywords:** 5’-DFUR, 5-fluorouracil, thymidine phosphorylase, prodrug, polymeric micelle, doxorubicin, SN-38

## Abstract

The compound 5’-deoxy-5-fluorouridine (5’-DFUR) is a prodrug of the anti-tumor drug 5-fluorouracil (5-FU). Thymidine phosphorylase (TP) is an enzyme that can convert 5’-DFUR to its active form 5-FU and the expression of TP is upregulated in various cancer cells. In this study, 5’-DFUR associated with amphiphilic copolymer poly(ε-caprolactone)-methoxy poly(ethylene glycol) (5’-DFUR-PCL-MPEG) was synthesized, characterized, and self-assembled into functional polymeric micelles. To demonstrate that the prodrug 5’-DFUR could convert into cytotoxic 5-fluorouracil (5-FU) by endogenous TP, HT-29 colorectal cancer cells were treated with 5’-DFUR-PCL-MPEG polymeric micelles for various time periods. Chemotherapeutic drugs doxorubicin (DOX) and 7-ethyl-10-hydroxycamptothecin (SN-38) were also encapsulated separately into 5’-DFUR-PCL-MPEG polymeric micelles to create a dual drug-loaded system. HT-29 cells were treated with DOX or SN-38 encapsulated 5’-DFUR-PCL-MPEG polymeric micelles to examine the efficacy of dual drug-loaded micelles. As a result, HT-29 cells treated with 5’-DFUR-PCL-MPEG polymeric micelles showed up to 40% cell death rate after a 72-h treatment. In contrast, HT-29 cells challenged with DOX or SN-38 encapsulated 5’-DFUR-incorporated polymeric micelles showed 36% and 31% in cell viability after a 72-h treatment, respectively.

## 1. Introduction

The compound 5-Fluorouracil (5-FU) is one of the main anti-tumor agents used to treat colon, breast and gastric cancers. Due to the short plasma half-life of 5-FU, it is often administered to patients through continuous infusion [1]. However, 5-FU is poorly tumor targeted and its treatment results in several severe adverse side effects in patients including intestinal discomfort, granulocytopenia, neuropathy, and cardiotoxicity [2,3,4]. Although some oral administrable 5-FU drugs have been developed [5], side effects such as mucositis and febrile neutropenia still remain. To circumvent toxicity, 5’-deoxy-5-fluorouridine (5’-DFUR), a prodrug of 5-FU, is commonly administered to patients [6]. 5’-DFUR is converted to its active and toxic form 5-FU through metabolic conversion by thymidine phosphorylase (TP), a gene overexpressed in many cancer types [7].

TP, also known as platelet-derived endothelial cell growth factor (PD-ECGF), is an angiogenic enzyme involved in pyrimidine nucleoside metabolism [8]. The reversible phosphorolytic cleavage of thymidine to thymine can be catalyzed by TP [9]. TP overexpressed in various tumors [10] significantly inhibits apoptosis of tumor cells [11]. The efficacy of 5’-DFUR is closely related to the level of TP expression and activity in tumor sites [12]. Several groups have exploited the enzyme-prodrug activation model of TP and 5’-DFUR and shown that the killing rate of cancer cell lines transfected with TP gene was augmented by the overexpressed TP level [13,14].

Clinically, 5’-DFUR has been used for the treatment of various tumor types [15,16,17]. Moreover, several studies have evaluated combination therapies of 5’-DFUR with other chemotherapy drugs such as tamoxifen, medroxyprogesterone acetate, and docetaxel with successful results [18,19,20]. While 5’-DFUR itself is slightly hydrophilic, intrinsic issues that are associated with other “free drugs”, such as poor solubility, unwanted toxicity, and short circulation times have propelled research into alternative drug delivery systems. There are several factors which contribute to the success of a synthesized therapeutic carrier. One must first address the physiochemical limitations of the drugs; second, one needs to address the biological hurdles in reaching the targeted tumorous tissue. Polymeric nanocarriers for drug delivery are being developed for a wide variety of cancers and chemotherapeutic drugs [21,22,23]. Specifically, several polymeric micellar carriers which house hydrophobic chemotherapy drugs doxorubicin (DOX), paclitaxel or 7-ethyl-10-hydroxycamptothecrin (SN-38) in their core are advancing into clinical trials with great success [24,25,26].

SN-38 is a biological metabolite of irinotecan hydrochloride (CPT-11). While CPT-11 is a prodrug which can be converted to SN-38 by carboxylesterases, SN-38 has shown to have 1000-fold more potent toxicity towards various cancer cells in vitro [27]. Moreover, metabolic conversion of CPT-11 to SN-38 in the liver and tumor has shown to be less than 10% of its original volume [28,29]. Therefore, direct use of SN-38 is advantageous for cancer therapy. Similarly, DOX is another widely used antitumor drug effective in the treatment of carcinomas of the breast, colon, thyroid and lung. However, due to the ensuing toxicity and low water solubility of free SN-38 and DOX, use of a drug carrier is warranted.

It has been shown previously that the hydroxyl groups on 5’-DFUR can be used for initiation in the ring-opening polymerization of ε-caprolactone to form 5’-DFUR-polycaprolactone (5’-DFUR-PCL) [30]. Poly(ε-caprolactone) (PCL) is commonly used for biomedical applications because of its excellent biodegradability and biocompatibility [31]. In this study, we further grafted hydrophobic 5’-DFUR-PCL with hydrophilic biodegradable methoxypolyethylene glycol (MPEG), widely used for drug delivery to form amphiphilic copolymers for micelle preparation [32,33,34]. Moreover, because camptothecin-based drugs are often used in conjunction with 5-FU as a first therapy [35], and the anticancer effectiveness of DOX, we encapsulated SN-38 and DOX respectively into our prodrug-incorporated polymeric micelles for an additive anticancer therapy.

To evaluate anticancer effectiveness of our synthesized polymeric micellar carriers, HT-29 colorectal cancer cells, which express endogenous TP, were treated with our micellar carrier. Moreover, SN-38 and DOX chemotherapeutic drugs were encapsulated individually into our synthesized micellar carriers and the additive effect of both 5’-DFUR and the chemotherapeutic drugs on HT-29 cell death was examined. Our results indicate that 5’-DFUR-PCL-MPEG micellar carriers are an effective and efficient means to deliver 5’-DFUR and chemotherapeutic drugs to tumor cells.

## 2. Materials and Methods

### 2.1. Materials

Epsilon-caprolactone (ε-CL), *N*,*N*’-dicyclohexyl carbodiimide (DCC), succinic anhydride, and pyrene were purchased from Acros Organics (Geel, Belgium). 5’-DFUR was obtained from TCI (Tokyo, Japan). Tetrahydrofuran (THF), toluene, dichloromethane (DCM), dimethyl sulfoxide (DMSO), deuterated dimethyl sulfoxide (DMSO-d_6_), CDCl_3_ with 1% tetramethylsilane (TMS), Sn(Oct)_2_, hexane, N-Hydroxysuccinimide (NHS), acetone, pyridine, magnesium sulfate, 2-propanol, methanol, hydrochloric acid (HCl), SN-38, DOX, methoxypolyethylene glycol amine (MPEG-NH_2_; MW = 5000), MPEG (MW = 350), penicillin-streptomycin, 0.25% trypsin/EDTA, RIPA lysis and extraction buffer, protease inhibitor, thymidine, thymine and 5-fluorouracil were all purchased from Sigma-Aldrich (St. Louis, MO, USA). HT-29 colorectal cell line was purchased from ATCC (HTB-38, Manassas, VA, USA). Fetal bovine serum (FBS) was purchased from Atlanta Biologicals (Flowery Branch, GA, USA). Dulbecco’s modified Eagles’ medium (DMEM) was purchased from Corning Cellgro (Manassas, VA, USA). Potassium phosphate and sodium hydroxide were purchased from Fisher Scientific (Fair Lawn, NJ, USA).

### 2.2. Characterization Methods

Gel permeation chromatography (GPC) was performed on a Waters binary HPLC pump equipped with a refractive index detector (Milford, MA, USA). Waters columns (styragel HR 3 (MW = 500–30,000) and HR 4E (MW = 50–100,000)) were installed in series. Molecular weight calibration was performed with polystyrene standards with MW ranged from 400 to 43,000. GPC analysis was performed by injecting 50 µL THF at a flow rate of 1 mL per min. Proton NMR spectra were obtained from a 400 MHz Varian Unity/Inova 400 (Sparta, NJ, USA). To gather FT-IR spectra by a FT-IR spectrometer (Jasco 4200, Tokyo, Japan), polymeric sample was first loaded onto a silicon wafer and THF was then added dropwise to dissolve the sample and evaporated afterwards to form a film for measurement.

### 2.3. Synthesis of 5’-DFUR-Incorporated Amphiphilic Polymers

A total of 50 mg of 5’-DFUR was weighed and mixed with 2.25 mL of ε-CL (2.25 mL) under a bath sonication for 5 min. The mixture was then added with 10 mg of Sn(Oct)_2_. The entire mixture was transferred into a 3-necked round bottom flask. The flask was purged with pure nitrogen and immersed in an oil bath for 24-h reaction at 140 °C. The final product was cooled to ambient temperature, dissolved in DCM, and precipitated by methanol at 4 °C. The precipitated product (i.e., 5’-DFUR-PCL) was further vacuum dried by a rotary evaporator at 40 °C. 0.5 mmol of 5’-DFUR-PCL and 1 mmol of succinic anhydride were weighed and dissolved in toluene in a 3-necked round bottom flask. After adding 1 mmol of pyridine, the solution was reacted at 70 °C for 48 h. The product was then precipitated by cold hexane and spun down. The pellet was re-dissolved in DCM and washed with 10% (*v/v*) HCl and saturated NaCl solution. The organic phase was isolated, dried with magnesium sulfate, and then filtered. The 5’-DFUR-PCL tagged with carboxylate group was recovered by precipitation in cold hexane, and then vacuum dried by rotovap. 0.54 mmol of 5’-DFUR-PCL-COOH and 2.7 mmol of NHS were mixed with 15 mL DCM. After adding 2.7 mmol of DCC, the reaction was run for 24 h at ambient temperature under nitrogen purging. After vacuum filtration, the filtrate was loaded with 35 mL cooled diethyl ether to get precipitate. After centrifugation, the collected pellet was washed with 2-propanol and then vacuum dried by rotovap to obtain 5’-DFUR-PCL-NHS. 10 mg of 5’-DFUR-PCL-NHS and 10 mg of MPEG-NH_2_ were weighed and dissolved by 20 mL DCM in a flask. The solution was reacted for 24 h under nitrogen environment. The solution was then dialyzed (molecular weight cutoff = 6–8 kD, Spectra/Por, Rancho Dominguez, CA, USA) to remove residual MPEG-NH_2_. The solution remained in the dialysis bag was vacuum dried by rotovap to obtain 5’-DFUR-PCL-MPEG.

### 2.4. Preparation of Polymeric Prodrug Micelles

A total of 10 mg of 5’-DFUR-incorporated amphiphilic polymer, with or without 0.2 mg DOX or 0.1 mg SN-38, were dissolved in 2 mL acetone under a sonication bath. The mixture was added dropwise into 10 mL deionized (DI) water to form polymeric prodrug micelles with or without the chemotherapeutic drug used. Acetone in the water phase was removed by rotovap and the final solution containing micellar particles was collected by flowing through a 0.45-µm filter.

### 2.5. CMC Determination

The critical micelle concentration (CMC) was determined by using fluorescent pyrene [36]. Briefly, 1 mg/mL of polymeric prodrug micelle was formed. Various amounts of DI water and polymeric prodrug micelle solutions were added respectively to glass vials to obtain micellar concentrations ranging from 5 × 10^−7^ to 1 mg/mL. Pyrene in acetone was then added separately to the prepared glass vials to obtain 6.0 × 10^−7^ mg/mL of pyrene in water, which is slightly lower than the saturation solubility [37]. The solutions were then kept for 8 h to reach equilibrium. Fluorescent spectra were measured by a Synergy MX spectrofluorometer (BioTek Instruments Inc., Winooski, VT, USA) with an excitation wavelength of 334 nm.

### 2.6. Size and Morphology of 5’-DFUR-Incorporated Polymeric Micelles

The average particle size of polymeric prodrug micelles was determined by a dynamic light scattering (DLS) instrument (Zetasizer Nano ZS, Malvern Instruments, Worcestershire, UK). The zeta potential of polymeric prodrug micelles dispersed in DI water was determined with a zeta potential analyzer (Zetasizer Nano ZS). Transmission electron microscopy (TEM) image of polymeric prodrug micelles was taken by JEM-4000FX (JEOL, Tokyo, Japan) at 80 kV. The TEM samples were prepared by adding 10 µL of polymeric prodrug micelle solution (1 mg/mL) onto a Formvar grid for 5 min and wicking away solution in excess. The samples were negatively stained with 10 µL phosphotungstic acid solution (2 wt%).

### 2.7. Drug Loading Content and Entrapment Efficiency

To determine the drug loading content of 5’-DFUR per mg of polymeric prodrug micelle, the absorbance of 5’-DFUR-PCL-MPEG micelles at t = 0 and t = 72 h was examined (λ = 269 nm) and used to calculate the amount based on a standard calibration curve of 5’-DFUR ranging from 0.002 to 1.0 mg/mL. To obtain drug-loading content and entrapment efficiency of DOX or SN-38 encapsulated in polymeric micelles, the obtained polymeric micellar solutions were frozen and lyophilized by a freezer dryer (Freezone, Labconco, Kansas City, MO, USA) to obtain dried nanoparticle product. The weighed nanoparticles were dissolved in DMSO/chloroform (1:1, *v/v*) and the absorbance of the solutions read at 485 nm (DOX) or 366 nm (SN-38) by a Synergy MX spectrofluorometer (BioTek Instruments Inc., Winooski, VT, USA). The weight of the entrapped drug was calculated by a calibration curve from 0.01 to 1 mg mL^−1^. Drug-loading content and entrapment efficiency were determined by Equations (1) and (2), respectively:

(1) $$\mathrm{Drug}\;\mathrm{loading}\;\mathrm{content}\;\left(\%\right)=\frac{\mathrm{weight}\;\mathrm{of}\;\mathrm{drug}\;\mathrm{in}\;\mathrm{nanoparticles}}{\mathrm{weight}\;\mathrm{of}\;\mathrm{nanoparticles}}\times 100$$

(2) $$\mathrm{Entrapment}\;\mathrm{efficiency}\;\left(\%\right)=\frac{\mathrm{weight}\;\mathrm{of}\;\mathrm{drug}\;\mathrm{in}\;\mathrm{nanoparticles}}{\mathrm{weight}\;\mathrm{of}\;\mathrm{drug}\;\mathrm{fed}\;\mathrm{initially}}\times 100$$

### 2.8. Drug Release Profiles

Polymeric prodrug micelles at a concentration of 1 mg/mL were prepared in phosphate buffered saline (PBS) 37 °C. Two mL of solution was placed in a dialysis tube (Float-A-Lyzer, molecular weight cutoff = 3.5–5 kD, Spectra/Por). The dialysis tube was then immersed in 50 mL PBS at ambient temperature and 37 °C with and without esterase (3 units/2 mL) to mimic cellular conditions. At specified time intervals, 5 µL of sample was removed and replaced with fresh PBS. The amount of 5’-DFUR released was analyzed by a Synergy MX spectrofluorometer at λ = 269 nm.

### 2.9. TP Activity Assay

HT-29 cells were cultured in DMEM supplemented with 10% FBS and 1% penicillin-streptomycin and incubated at 5% CO_2_ balanced chamber controlled at 37 °C with humidified air. 5 × 10^5^ cells were suspended by treating the cells with 0.25% trypsin/EDTA and collected by centrifugation at 400× *g* for 5 min. The cell pellet was washed by 1× PBS, and cells were re-suspended in 100 µL RIPA buffer supplemented with 1% protease inhibitor. The cell suspension was placed on ice for 5 min, followed by tip sonication for 10 s 3 times with 30 s interval on ice using a tip sonicator (Misonix XL-2000, Farmingdale, NY, USA). The cell suspension was kept on ice for additional 15 min, and then centrifuged at 14,000× *g* for 15 min to collect cell lysate. 15 µL of HT-29 cell lysate were added to a 40-µL reaction mixture containing 25 mM potassium phosphate buffer (pH 7.4) and 10 mM thymidine or 5’-DFUR. The mixture was incubated at 37 °C for 4 h. The reaction was terminated by the addition of 200 µL of ice-cold 500 mM NaOH. The absorbance readings at 300 nm for thymine (converted from thymidine) and 305 nm for 5-FU (converted from 5’-DFUR) were measured respectively [13] by a SpectraMax M2e Microplate Reader (Molecular Devices, Sunnyvale, CA, USA). The amount of thymine or 5-FU generated in the reaction mixture was calculated using the thymine or 5-FU calibration curve. The total protein amount of the cell lysate was determined by the Bradford assay. The TP activity was expressed as the amount of thymine or 5-FU (µmol) converted/mg protein/h.

### 2.10. Cytotoxicity Studies

Human colorectal HT-29 cells were seeded in 24-well plates containing 0.5 mL DMEM supplemented with 10% FBS and 1% penicillin-streptomycin and cultivated for 24 h at 37 °C incubator maintained at 5% CO_2_ balanced with humidified air. In one well, 1 mL of 2 mg mL^−1^ of polymeric micelle solution was added. Serial dilutions were preformed to a final concentration of 0.25 mg/mL. After 72-h incubation, cell viability was assessed using the MTT assay; 200 µL of 4 mg/mL MTT solution was loaded into the culture wells and incubated for 4 h. The medium containing unreacted MTT was disposed and 300 µL DMSO was added to dissolve the insoluble purple formazan crystals formed. The absorbance at 590 nm was measured by a Synergy MX spectrofluorometer. The percentage of cell viability calculated against the control group without polymeric micellar challenge will be determined.

## 3. Results and Discussion

### 3.1. Synthesis and Characterization of Amphiphilic Prodrug Polymers

**Figure 1:**
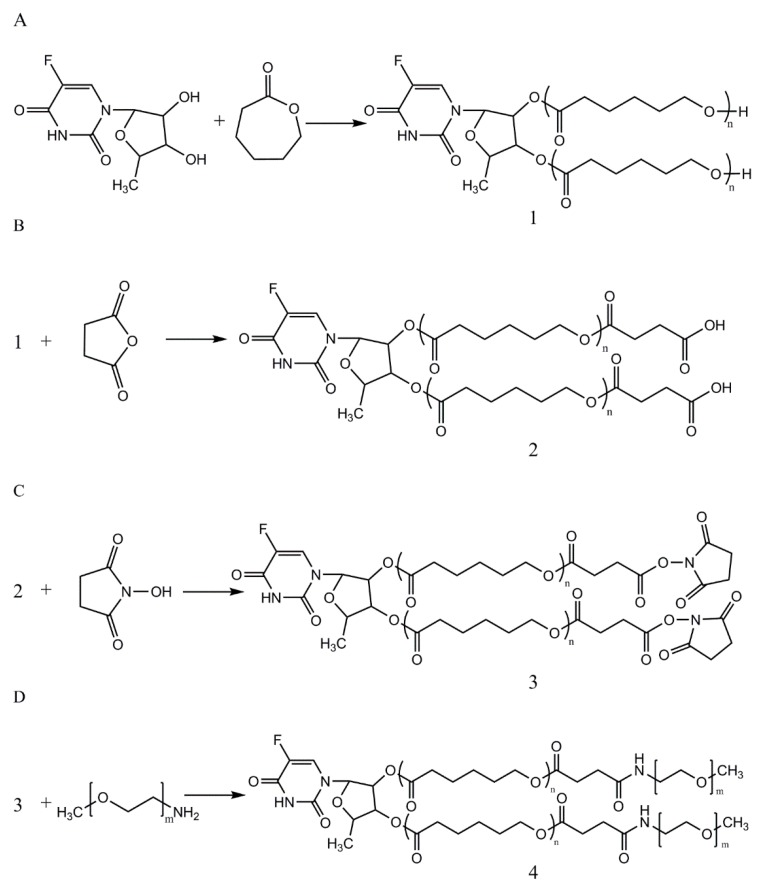
Synthetic scheme of (**A**) 5’-DFUR-PCL, (**B**) 5’-DFUR-PCL-COOH, (**C**) 5’-DFUR-PCL-NHS, and (**D**) 5’-DFUR-PCL-MPEG.

**Figure 2:**
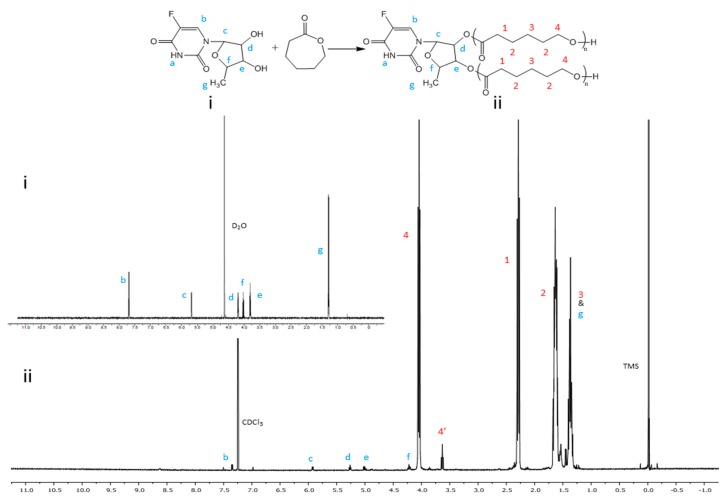
^1^H NMR spectra of (**i**) 5’-DFUR and (**ii**) 5’-DFUR-PCL.

Figure 1A–D illustrates the preparation of 5’-DFUR-PCL-MPEG copolymer. First, 5’-DFUR-PCL was synthesized directly via the ring-opening polymerization of ε-CL initiated by 5’-DFUR as previously reported [30]. ^1^H NMR spectra of prodrug 5’-DFUR and 5’-DFUR-PCL post-synthesis are shown respectively in Figure 2 (i) and (ii). Characteristic resonance peaks associated with 5’-DFUR including δ = 1.43 (g-CH_3_), 4.20 (f-CH), 5.02 (e-CH), 5.25 (d-CH), 5.95 (c-CH), and 7.97 (b-CH) ppm were seen in synthesized 5’-DFUR-PCL. Chemical shifts associated with PCL were seen at 1.40 (3-CH_2_), 1.65 (2-CH_2_), 2.34 (1-CH_2_), 3.62 (4’-CH_2_), and 4.05 (4-CH_2_) ppm. These spectra demonstrated evidence of 5’-DFUR acting as initiator for the ring-opening polymerization of ε-CL. GPC data concerning the polymerization of PCL by 5’-DFUR is given in Table 1, accordingly, the number average molecular weight (M_n_) of 5’-DFUR-PCL was approximately 15 kDa with a polydispersity index (PDI) of 1.24.

**Figure 3:**
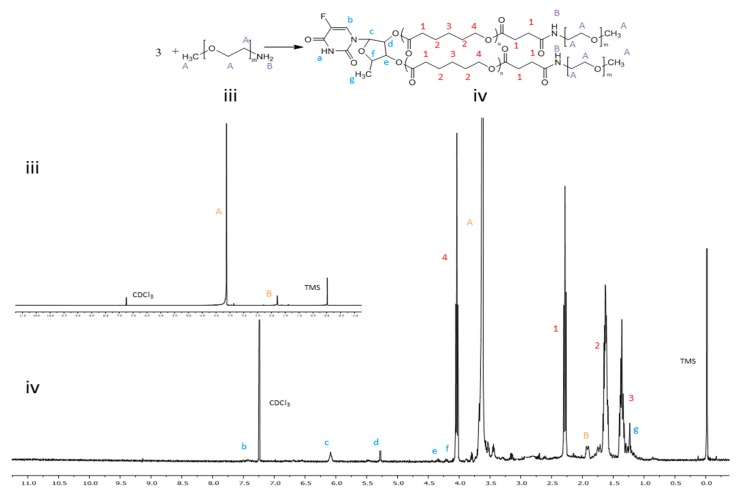
^1^H NMR spectra of (**iii**) MPEG and (**iv**) 5’-DFUR-PCL-MPEG.

Hydrophobic 5’-DFUR-PCL was further grafted with MPEG (MW = 5000) as shown in Figure 1B–D. GPC analysis revealed that 5’-DFUR-PCL-MPEG amphiphilic copolymer had a M_n_ of 28 kDa and PDI of 1.19 (Table 1). Figure 3 (iii) and (iv), shows the ^1^H NMR of MPEG-NH_2_ and 5’-DFUR-PCL-MPEG. The peaks at 3.63 (A-OCH_2_) belonged to MPEG can be observed in Figure 3 (iv). Since conjugation of MPEG to 5’-DFUR-PCL is through the amide linkage (Figure 1D), the change of the peak at 1.79 (B-NH_2_) from a singlet in Figure 3 (iii) to a multiplet in Figure 3(iv) confirms MPEG was bound to 5’-DFUR-PCL. Further verification of MPEG grafted to 5’-DFUR-PCL was done by FT-IR analysis.

**Figure 4:**
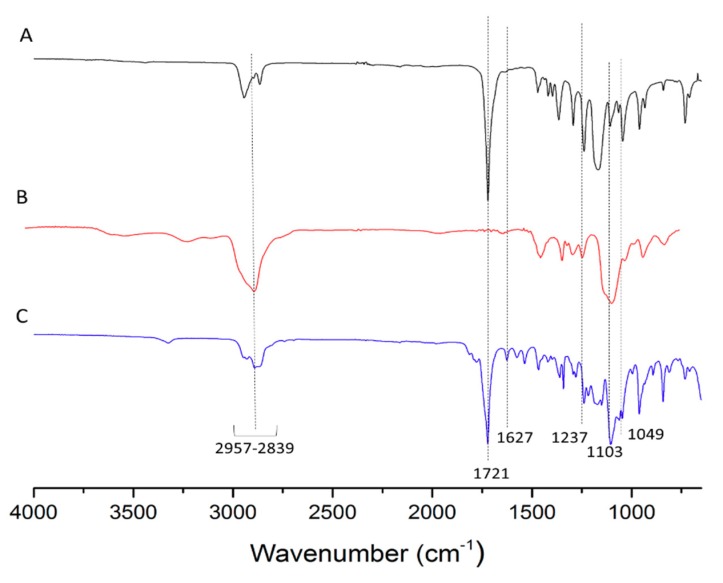
FT-IR spectra of (**A**) 5’-DFUR-PCL, (**B**) MPEG-NH_2_, and (**C**) 5’-DFUR-PCL-MPEG.

FT-IR was used to show the grafting of MPEG to 5’-DFUR-PCL-NHS (Figure 1D). FT-IR spectra of 5’-DFUR-PCL (A), MPEG-NH_2_ (B) and 5’-DFUR-PCL-MPEG (C) are shown in Figure 4. C-H stretching vibrations are observed from 2957–2839 cm^−1^ for all samples. FT-IR absorption peaks of PCL and MPEG at 1721 cm^−1^ attributed to the C=O stretching and at 1103 cm^−1^ of the C-O-C, respectively, were seen in the FT-IR spectra. These peaks, as well as characteristic peaks contributed from 5’-DFUR such as C-F stretching at 1049 cm^−1^, C-N stretching of the primary and secondary aromatic amine at 1627 and 1237 cm^−1^ were all found in Figure 4A,C, showing successful initiation of polymerization of ε-CL by 5’-DFUR.

### 3.2. Formation and Characterization of 5’-DFUR-Incorporated Polymeric Micelles

**Figure 5:**
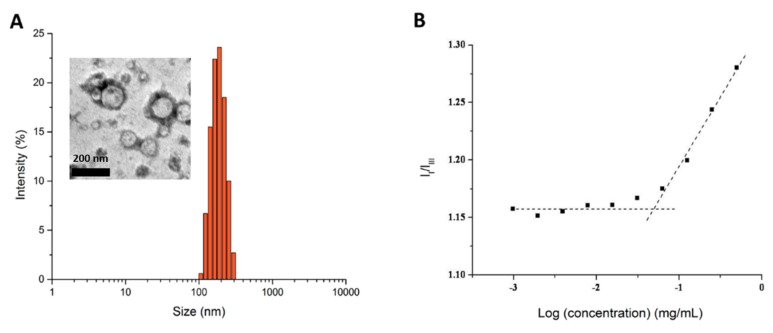
(**A**) Particle size distribution, and (**B**) plot of the intensity ratio (I_I_/I_III_) versus concentration of 5’-DFUR-PCL-MPEG micelles. Inset represents a TEM image. Scale bar denotes 200 nm.

It is known that amphiphilic polymers can self-assemble into micelles in selected solvents. The amphiphilic 5’-DFUR-PCL-MPEG copolymer used in this study, could self-assemble into micelles in aqueous solution by the solvent-evaporation method. Here, despite 5’-DFUR being slightly water soluble, 5’-DFUR-PCL was the hydrophobic core segment and MPEG was the hydrophilic outside shell. In control polymeric micelles, MPEG_350_-PCL was the hydrophobic core segment and MPEG was the hydrophilic segment. The reason of selecting MPEG_350_ as the initiator for control studies was due to the fact that MPEG_350_ has a molecular weight close to 5’-DFUR’s (MW = 246.19 g/mol). The average size of 5’-DFUR-incorporated polymeric micelles and control micelles with and without encapsulating DOX or SN-38 and zeta potential as determined by dynamic light scattering (DLS) and zetasizer are shown in Table 2. The size of 5’-DFUR-PCL-MPEG micelles was around 220.5 nm with a standard deviation of 41.7 and a zeta potential of 1.2 mV due to MPEG’s neutral change (Figure 5A). Based on dynamic light scattering, the micelles have a polydispersity index (PDI) of 0.22. The PDI was not surprising as a PDI of 0.3 and below is considered acceptable for lipid-based carriers and indicates a relatively monodisperse sample [37]. Micelles were also characterized by TEM analysis as shown in the Figure 5 insert. The TEM images showed the polymeric prodrug micelles have an average size of ~150 nm which is slightly lower than the results from DLS, due to the hydrodynamic radius of particles determined by the dynamic light scattering. As shown in Table 2, encapsulation of DOX or SN-38 did not affect the particle size substantially with an average size of 167.5 and 267.5 nm, respectively.

The CMC of 5’-DFUR-PCL-MPEG was determined using pyrene as a hydrophobic florescent probe to confirm the formation of micellar structures [38]. Figure 5B reveals the CMC value of 5’-DFUR-incorporated polymeric micelles in aqueous solution. The intensity ratio of the first and third vibrational bands (I_338_/I_335_) against polymer concentration (Log(concentration)) in pyrene excitation spectra was plotted. A flat region in the low concentration extreme and sigmoidal region in the crossover region was determined, and the CMC of 5’-DFUR-incorporated micelles was 56 mg L^−1^.

### 3.3. Evaluation of Drug Loading Content and Entrapment Efficiency

Table 2 summarizes the drug loading content and entrapment efficiency of 5’-DFUR, DOX, and SN-38 in both 5’-DFUR-incorporated polymeric micelles and control micelles. To determine the percentage of drug loading of 5’-DFUR per mg of micelle formulation, the absorbance of 5’-DFUR before (t = 0 h) and after drug release (t = 72 h) was investigated and the amount was calculated from a standard calibration curve of 5’-DFUR. It was found that 5’-DFUR comprised 9.8% of 5’-DFUR-PCL-MPEG micelles. Due to the hydrophobic nature of SN-38, it was surmised that the extent of drug loading and entrapment would be high. As can be seen in Table 2, our results showed that the drug loading content and entrapment efficiency of SN-38 in 5’-DFUR-PCL-MPEG and control micelles was 3.4% and 86.3% and 3.9% and 97.6%, respectively. In contrast, DOX which is slightly hydrophilic had a lower encapsulation efficiency at 68.8% and 65.6% in prodrug-incorporated polymeric micelles and control micelles, respectively. However, due to the fact that more DOX was used for encapsulation, the drug loading content was higher at 10.8% and 10.4% Drug encapsulation efficiency is an important design parameter in the development of therapeutic nanocarriers. An ideal nanocarrier should have a high drug encapsulation efficiency and small size to evade the mononuclear phagocyte system. The synthesized 5’-DFUR-incorporated micelles developed here exhibit both of these qualities.

### 3.4. TP Activity Assay

The expression level of TP is closely related to the efficiency of 5-FU conversion. High TP expression levels has been found in some tumor tissues like stomach, colon and ovary tumor sites [39]. The expression of TP has been confirmed in various cancer cell lines such as HT-29 [40,41], SKBR3 [42], A431 [43] and A549 [43]. On the other hand, the level of TP expression may vary from different colon cancer cell lines [44]. For example, no endogenous TP is found in COLO320, and the expression level on RT112 is low [45]. In the current study, TP-containing HT-29 cell lysate was used to convert thymidine to thymine, and the thymine formed was around 2.89 µmol/mg protein/h (Table 3). This data suggests a high level of TP enzyme activity in HT-29 cells, compared to wild type breast cancer cell MCF-7 with low enzyme activity of 38 nmol/mg protein/h [13]. Furthermore, cytotoxic 5-FU converted from prodrug 5’-DFUR reacted with HT-29 cell lysate revealed TP activity of ~2.21 µmol/mg protein/h (Table 3) which is in line with the enzyme activity aforementioned by the conversion of thymidine to thymine. Our results indicated that TP expression and activity in HT-29 cells are high enough to convert 5-FU from loaded 5’-DFUR, thereby resulting in eradication of cancer cells. 

### 3.5. In Vitro Drug Release

**Figure 6:**
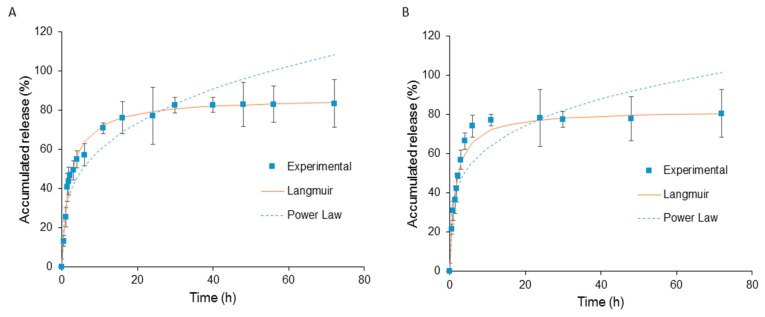
In vitro drug release profile of 5’-DFUR from 5’-DFUR-PCL-MPEG micelles in PBS at 37 °C (**A**) with esterase, (**B**) without esterase (mean ± SD, *n* = 3).

The in vitro release behavior of 5’-DFUR both at 37 °C with and without esterase was studied and the results are shown in Figure 6A,B, respectively. To mimic cellular conditions, esterase at a concentration of 3 units/2 mL was chosen [46]. A two-phase release profile was observed in all conditions with a 2-h initial burst release followed by continuous release pattern up to 72 h. It was found that it required 2 h before esterase was able to increase the release of 5’-DFUR. This observation is in line with other researchers due to the fact that esterase has to take time to diffuse into the micelle and to activate [47,48]. The release of 5’-DFUR in samples without esterase was caused by hydrolysis of ester linkage between 5’-DFUR and PCL. It is surmised that the sustained release of 5’-DFUR compared to previously reported acyclovir prodrug release is due to that fact that polymerization can be initiated at two hydroxyl points rather than one from acyclovir [49]. Accumulative release of each sample reached a maximum between 78–86%.

The release of 5’-DFUR from 5’-DFUR-PCL-MPEG was modeled using both Power Law and Langmuir models as shown in Figure 6A,B. The Power Law model was not a good fit for the release of 5’-DFUR from polymeric prodrug micelles. Here, we obtained an exponent, *n*, value equal to 0.24 and 0.30 with and without esterase, respectively. If *n* is 0.43, for a sphere, this would indicate Fickian diffusion [50]. Due to the fact that our release is not solely though diffusion (i.e., 5’-DFUR is chemically bound to PCL though ester bond), our release is reaction diffusion. Here, the prodrug 5’-DFUR, is released through hydrolysis (Figure 6B) or a combination of hydrolysis and esterase (Figure 6A). Therefore, we chose to also model our data with the Langmuir model. The Langmuir model is an enzyme kinetics model, and as can be seen in Figure 6, a good fit for the release of 5’-DFUR from polymeric prodrug micelles. The dissociation constant (K_d_) for the release of 5’-DFUR from 5’-DFUR-PCL-MPEG was found to be 1.48 and 3.07 with and without esterase, respectively.

**Figure 7:**
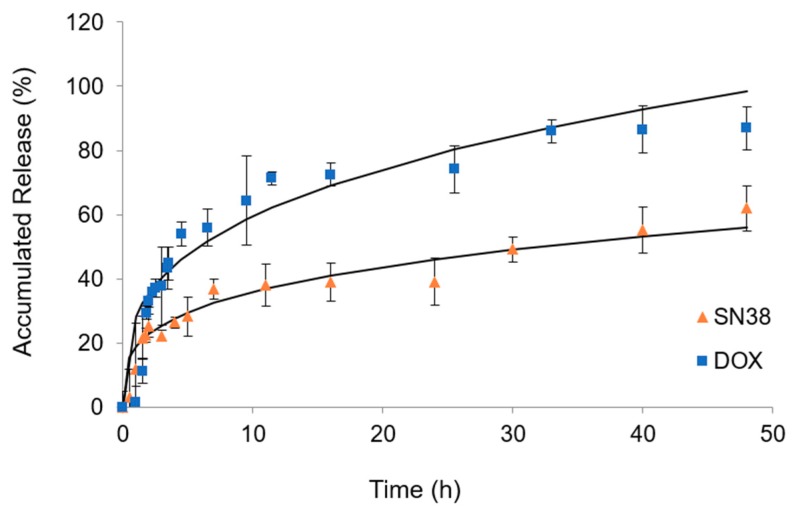
In vitro drug release profiles of DOX and SN-38 from 5’-DFUR-PCL-MPEG micelles in PBS at 37 °C (mean ± SD, *n* = 3).

The release of DOX and SN-38 at 37 °C in 5’-DFUR-PCL-MPEG is shown in Figure 7. The release profiles showed that a cumulative release of DOX and SN-38 from 5’-DFUR-incorporated micelles in PBS at 37 °C was up to 87% and 62%, respectively, after 48 h. Moreover, we modeled the data using the Power Law and it was found to be a good fit for both of the encapsulated chemotherapeutic drugs. For the release of DOX and SN-38, we obtained *n* values of 0.41 and 0.43. These values are very close to the exponent value *n* (0.43) for the Fickian diffusion of a sphere, indicating that Fickian diffusion is most likely the release mechanism for encapsulated chemotherapy drugs DOX and SN-38.

### 3.6. Cytotoxicity of Polymeric Prodrug Micelles Loaded with Chemotherapeutic Drugs

**Figure 8:**
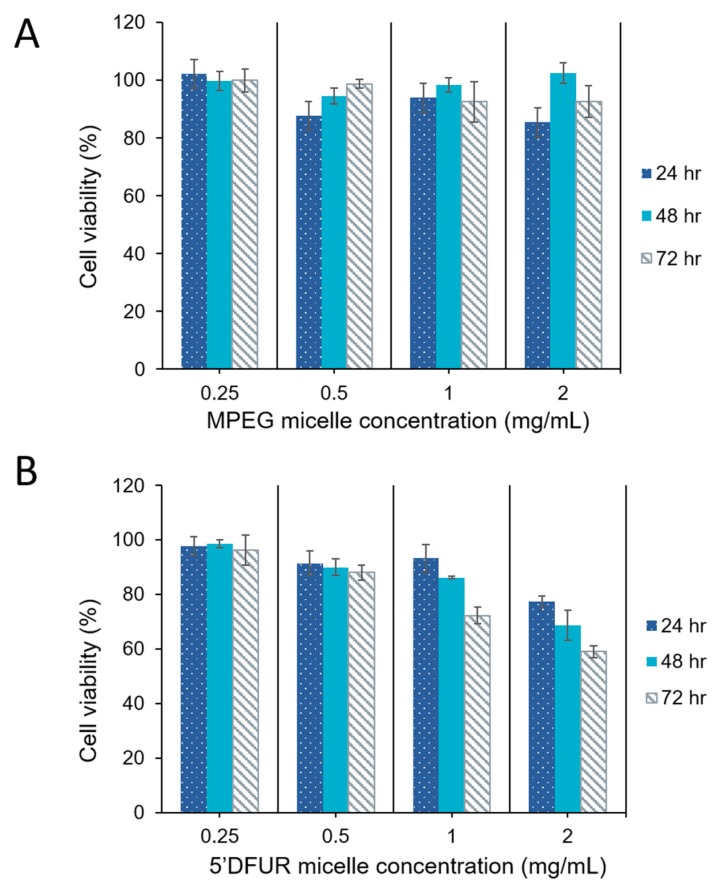
Cell viability of HT-29 cells after treatment with polymeric micelles for 24, 48, and 72 h. (**A**) MPEG_350_-PCL-MPEG polymeric micelles, (**B**) 5’-DFUR-PCL-MPEG polymeric micelles (mean ± SD, *n* = 3).

In vitro toxicity of polymeric prodrug micelles to parental HT-29 cells was evaluated. Due to the fact that HT-29 cells express endogenous TP levels [39], 5’-DFUR released from micelles would be converted to its active and toxic form 5-FU. As discussed with respect the drug release profile, 5’DFUR is released by reaction diffusion. Therefore, it is surmised that 5’-DFUR is released from the micellar carriers through both diffusion at the surface of the cell as well as through endocytosis of the micellar carriers. The internalized drug-loaded micellar carriers upon reaching the lysosome will be degraded which will release more of the prodrug. The internalized prodrug is then converted via endogenous TP to the toxic drug 5-FU. Figure 8 compares the viability of MPEG_350_-PCL-MPEG control micelles and 5’-DFUR-PCL-MPEG prodrug micelles. Here, it can be seen that micelles without prodrug are nontoxic up to a concentration of 2 mg mL^−1^ (Figure 8A). In contrast, the viability of HT-29 cells was decreased to 60% when challenged with 2 mg mL^−1^ 5’-DFUR-PCL-MPEG micelles for 72 h (Figure 8B). In prior work, it has been shown that HT-29 cells treated with polymeric micelles tagged with acyclovir (ACV) [49] or ganciclovir (GCV) [51] did not exhibit apparent toxicity. This is because ACV and GCV prodrugs are not converted to their active and toxic form by TP. Cell viability results clearly show that cell death occurs due to the conversion of 5’-DFUR to its active and toxic form 5-FU by endogenous TP within the HT-29 cells. Moreover, results indicated that a concentration of 5’-DFUR-incorporated micelle greater than or equal to 0.5 mg mL^−1^ is needed for cell death to occur.

**Figure 9:**
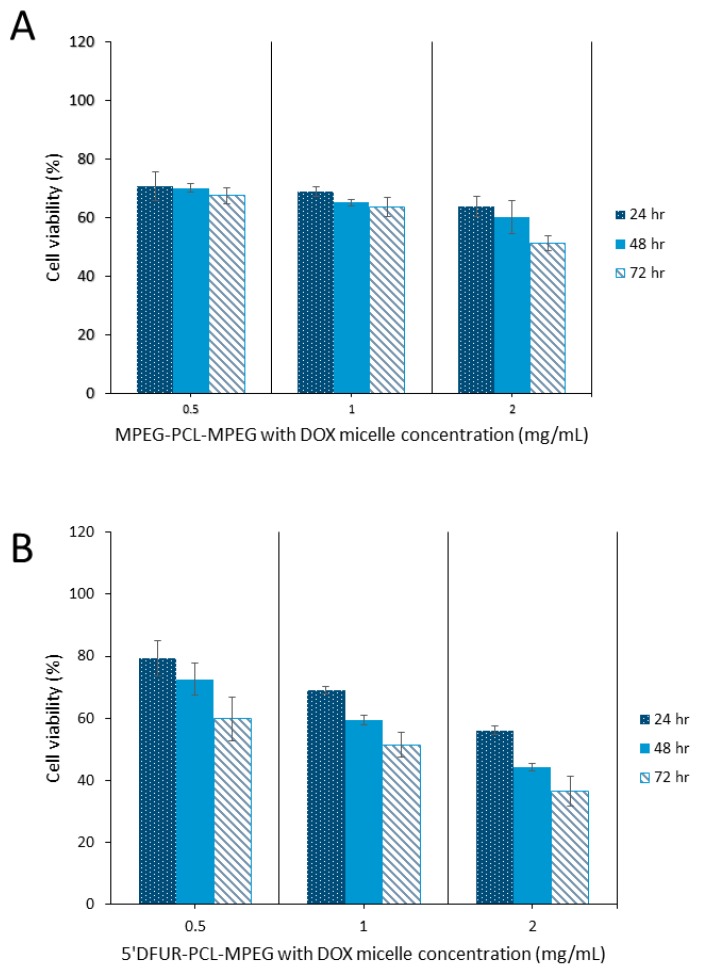
Cell viability of HT-29 cells after treatment with polymeric micelles encapsulating DOX for 24, 48, and 72 h. (**A**) MPEG_350_-PCL-MPEG polymeric micelles, (**B**) 5’-DFUR-PCL-MPEG polymeric micelles (mean ± SD, *n* = 3).

**Figure 10:**
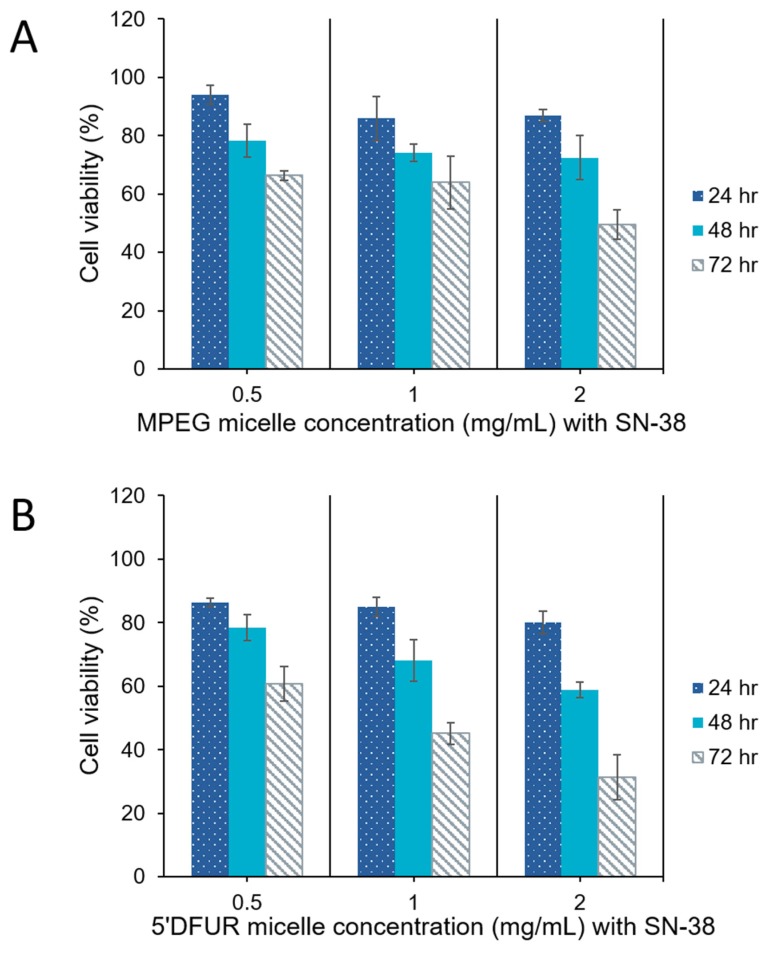
Cell viability of HT-29 cells after treatment with polymeric micelles encapsulating SN-38 for 24, 48, and 72 h. (**A**) MPEG_350_-PCL-MPEG polymeric micelles, (**B**) 5’-DFUR-PCL-MPEG polymeric micelles (mean ± SD, *n* = 3).

As can be seen from Figure 9 and Figure 10, HT-29 cell viability was decreased in both control (A) and prodrug-incorporated micelles with encapsulated chemotherapy drug (B). Control micelles without chemotherapy drug showed little to no toxicity (Figure 8A). after the encapsulation of DOX or SN-38 into MPEG_350_-PCL-MPEG micelles, viability was reduced to 53% and 43%, respectively, with the highest dose for 72 h (Figure 9A and Figure 10A). Toxicity of HT-29 cells treated with 5’-DFUR-PCL-MPEG micelles with encapsulated DOX showed an increased cell death from 60% viability without DOX to 36.6% viability (Figure 9B). This corresponds to an additive effect from both 5’-DFUR enzymatically catalyzed to 5-FU by TP endogenously expressed in HT-29 cells and DOX chemotherapy drug. The toxicity of 5’-DFUR-incorporated polymeric micelles which encapsulated SN-38 was also increased from 60% cell viability to 31%; again, showing an additive effect between 5’-DFUR and chemotherapy drug SN-38 in cancer cell treatment. Wang et al. have loaded poly(lactide-co-glycolide) (PLGA) nanoparticles with 5-FU [52]. In their study, ~70% of HT-29 cells were killed when cells were treated with PLGA nanoparticles loaded with 50 µg/mL 5-FU. The efficacy of 5’-DFUR-incorporated polymeric micelles encapsulated with SN-38 was comparable to 5-FU loaded PLGA nanoparticles. In another study, HT-29 cells were treated with magnetic particles loaded with cathelicidin LL-37 [53]. As a result, LL-37 loaded magnetic particles caused 50% drop in cell viability. In short, the results of cell viability show that delivery of 5’-DFUR-incorporated polymeric micelles can decrease viability of HT-29 cells, and that encapsulation of chemotherapy drugs can substantially increase cell death.

## 4. Conclusions

The results of this study show that amphiphilic copolymer 5’-DFUR-PCL-MPEG was successfully synthesized and characterized, and its polymeric micelles were fabricated and analyzed. Our results further demonstrated that the formed polymeric prodrug micelles could successfully deliver and release prodrug 5’-DFUR into HT-29 colorectal cancer cells via the hydrolysis of ester linkage between 5’-DFUR and PCL. Moreover, it was clearly shown that the amount of endogenous TP expressed in HT-29 colorectal cancer cells is sufficient enough to convert prodrug 5’-DFUR into cytotoxic 5-FU, thereby killing HT-29 cells. In addition, co-delivery of 5’-DFUR and DOX or SN-38 greatly enhanced malignant cell death.

## Figures and Tables

**Table 1:** Characterization of 5’-DFUR-PCL-MPEG and MPEG_350_-PCL-MPEG amphiphilic copolymer.

Sample	M_w_ (Da)	M_n_ (Da)	Polydispersity (M_w_/M_n_)
5’-DFUR-PCL	18,796	15,158	1.24
5’-DFUR-PCL-MPEG	33,927	28,510	1.19
MPEG_350_-PCL	24,600	17,053	1.44
MPEG_350_-PCL-MPEG	19,415	21,624	1.36

**Table 2:** Characteristics and drug loading of 5’-DFUR-incorporated polymeric micelles.

Sample	Size (nm)	Zeta (mV)	Drug Loading Content (%)	Entrapment Efficiency (%)
5’-DFUR-PCL-MPEG	220.5	1.23	--	--
5’-DFUR-PCL-MPEG (DOX)	167.5	−0.11	10.8	68.8
5’-DFUR-PCL-MPEG (SN-38)	267.5	1.01	3.4	86.3
MPEG_350_-PCL-MPEG	202.5	0.74	--	--
MPEG_350_-PCL-MPEG (DOX)	222	2.15	10.4	65.6
MPEG_350_-PCL-MPEG (SN-38)	148	1.21	3.9	97.6

**Table 3:** TP activity of HT-29 cell lysate, with regard to thymidine and 5’-DFUR phosphorolytic cleavage.

Cell Line	Thymine Released (µmol/mg protein/h)	5-FU Released (µmol/mg protein/h)
HT-29	2.89 ± 0.27	2.21 ± 0.12
